# Investigation of the usefulness of zaleplon at two doses to induce afternoon-sleep under noise interference and its effects on psychomotor performance and vestibular function

**DOI:** 10.1186/s40779-016-0075-4

**Published:** 2016-03-01

**Authors:** Liang-En Chen, An-Dong Zhao, Qing-Jun Zhang, Feng Wu, Zhao-Li Ge, Hua Ge, Hao Zhan

**Affiliations:** Department of Pharmacology, Institute of Aviation Medicine, Air Force, Beijing, 100142 China

**Keywords:** Insomnia, Sleep, Hypnotic, Zaleplon, Psychomotor performance, Vestibular function

## Abstract

**Background:**

Military operation personnel often suffer from sleep difficulty because of their work requirements. In this study, we investigated the efficacy of zaleplon at two doses to induce afternoon-sleep under noise interference and its effects on psychomotor performance and vestibular function; we subsequently established the optimal dosage regimen for military operation personnel.

**Methods:**

Twenty-two healthy young male volunteers were recruited for the study. Eight subjects took 10 mg or 15 mg of zaleplon and placebo alternately and then were exposed to noise. Changes in polysomnography (PSG) indices, including sleep latency (SL), sleep efficiency (SE) and sleep structure, were recorded after drug administration. After awakening, the volunteers’ subjective judgments of sleep quality and sleepiness were measured. Eight volunteers underwent 3 psychomotor performance tests at a one-week interval, and the psychomotor performance tests were conducted before and after taking zaleplon and placebo. Six volunteers participated in the vestibular function test session, and parameters, including optokinetic nystagmus (OKN), vestibular ocular reflex (VOR), visual-vestibular ocular reflex (VVOR) and vestibular ocular reflex fixation suppression (VOR-Fix), were detected by the same experimental design as described above. The data of sleep observations were subjected to one-way variance analysis.

**Results:**

Compared with the placebo group, SL was shortened significantly, and the scores of subjective sleep quality and sleep depth were clearly increased in the zaleplon 10 mg group (*P* < 0.05). Moreover, the SE and the percent of REM (rapid eye movement) sleep were increased remarkably in the zaleplon 15 mg group (*P* < 0.01). Furthermore, the SE, percent of REM sleep and scores of subjective sleep depth in the zaleplon 15 mg group were significantly higher than in the zaleplon 10 mg group (*P* < 0.05). The psychomotor performance did not change significantly after ingestion of 10 mg or 15 mg of zaleplon, whereas the OKN and VOR gains were lower in the two dose groups of zaleplon (*P* < 0.05) and restored to normal 3 h after drug ingestion.

**Conclusion:**

Zaleplon is an ideal hypnotic for military personnel, and its hypnotic efficiency is dose-related under noise interference; a 15 mg dose of zaleplon could provide significantly better sleep than a 10 mg dose of zaleplon.

## Background

In military operations, it is common to change work schedules and work places [1]. For example, during the Persian Gulf War, many military units underwent short notice transmeridian deployment with immediate commencement of 24 h operations upon arrival. Some of these individuals likely suffered from circadian desynchronosis, which blunted their effectiveness [2]. Therefore, the United Sates Air Force approved the limited use of one short acting hypnotic medication to relieve the sleep disorders of the aircrew on the battlefield and one stimulant medication to enhance the alertness of these individuals [3].

Zaleplon is a pyrazolopyrimidine compound that selectively binds to the γ-aminobutyric acid (GABA) A receptor complex. Zaleplon is rapidly absorbed with peak plasma concentrations at approximately 1 h and is rapidly eliminated with a plasma elimination half-life of approximately 1 h [4, 5]. Zaleplon is commonly administered at 10 mg oral doses, although various experiments have shown some differences in the sleep-inducing effects of zaleplon. Whitmore et al. [6] found that compared with placebo, zaleplon at a 10 mg oral dose could increase slow-wave sleep and the total sleep time in a normal sleep environment. Stone et al. [7] investigated the efficacy of zaleplon in promoting sleep in healthy volunteers with noise-induced sleep maintenance insomnia. The results showed that zaleplon at doses of 10 mg and 20 mg reduced the latency to persistent sleep, and the 20 mg dose reduced N1 of non-rapid eye movement (NREM) sleep compared with placebo. However, Simons et al. [8] observed the usefulness of temazepam (20 mg) and zaleplon (10 mg) to improve 4.5 h of sleep in the afternoon and showed that the use of zaleplon at the dose of 10 mg had no significant advantage over placebo. Therefore, this study was conducted to assess the usefulness of zaleplon at two dose levels (10 mg and 15 mg) to improve 4 h of sleep in the afternoon under strong noise interference, to compare the effects of zaleplon on psychomotor performance and vestibular function 1–8 h after drug administration and to establish the optimal dosage regimen for military operation personnel.

## Methods

### Subjects

Twenty-two healthy male volunteers (mean age, 22.0 years; range, 19–33 years) participated in the study. The subjects had no history of insomnia or smoking and were drug free for 3 months before the study. The medical ethics committee of the Institute of Aviation Medicine, Air Force, People’s Liberation Army, granted approval for the study. Informed consent was obtained from all subjects after a full explanation of the nature of the study. The subjects were paid for their participation.

### Assessment methods

A polysomnography (PSG) device (P&G9600, Beijing Xinxing Biomedical Electronic Engineering Center, Beijing, China) was used to record the objective sleep quality [9]. The following parameters of sleep process and sleep structure were calculated automatically: sleep latency (SL), sleep efficiency (SE), percentage of N1, N2, N3 of NREM sleep and rapid eye movement (REM) sleep. The quality of sleep after awakening was assessed by the subjective Sleep Quality Scale (SQS) and subjective Sleep Depth Scale (SDS). The results of the SQS and SDS are a quality score ranging from 1 (very poor) to 7 (very good). The Stanford Sleepiness Scale (SSS) was used to assess the subjective sleepiness after awakening [10]. The result of the SSS is a score with increasing sleepiness from 1 to 7.

The psychomotor performance tests included a vigilance and tracking dual task, choice reaction time (CRT) and critical flicker fusion (CFF) frequency detections. The vigilance and tracking dual task were performed on a computer and measured the correct rate of four digit addition and the correct rate of controlling the simulated flying state of an airplane and the combination of these tasks [11]. The CRT test was conducted to assess the recognition reaction time when red, green or yellow lights were randomly presented [12]. The individual CFF frequency was determined using the mean of two ascending and two descending presentations [12].

The computerized vestibular function examination apparatus was used to evaluate the vestibular function. This system was produced by Beijing Heping Medical Instrument Factory and operated by the Institute of Aviation Medicine. The vestibular function evaluation protocols include optokinetic nystagmus (OKN), vestibular ocular reflex (VOR), visual-vestibular ocular reflex (VVOR) and vestibular ocular reflex fixation suppression (VOR-Fix) [13]. During the process of the VOR and VOR-Fix tests, nystagmus was induced by sinusoidal angular rotation in horizontal plane at 0.05 Hz and a peak velocity of 60°/s. While the VVOR test was performed, nystagmus was induced by both sinusoidal angular rotation with a peak velocity of 60°/s in the horizontal plane at 0.05 Hz and by stripes on a wall. When the OKN test was conducted, OKN was induced by full-field sinusoidal moving visual stimuli at 0.05 Hz and a peak velocity of 60°/s. The gain in the nystagmus response during stimulation for three cycles was calculated by a computer.

### Design and treatments

For measuring the sleep-inducing effects, using the double-dummy technique, a single dose of 10 mg or 15 mg of zaleplon (Sibao Pharmaceutical Company, Wuhan, China) or placebo were randomly used in a double-blind crossover design. Between the drug administrations, there was a wash-out period of 7 days. A randomized, double-blind repeated-measures protocol was designed to assess the effect of zaleplon at two doses and placebo on psychomotor performance. To maintain the double-blind nature of this work, all medications were prepared in identical capsule format.

### Procedure

The volunteers were medically examined and instructed to abstain from the use of hypnotics or psychoactive drugs (tranquilizers) for 1 wk before any experimental session, and from alcohol, over-the-counter medications and caffeinated beverages within 12 h of each experimental session. The volunteers were instructed to maintain regular sleep/wake cycles during the study and to avoid strong sports. As they were exposed to the simulated noise environment (150 Hz, approximately 95 dB), eight volunteers took 10 mg or 15 mg of zaleplon and placebo alternately at 14:00 pm. PSG for 4 h after taking pills was recorded. After awakening, their subjective judgments of sleep quality and sleepiness were assessed.

Eight volunteers were trained to participate in the psychomotor performance tests. On the days of the tests, each subject performed a baseline test session at 11:00 am. The subjects were tested for psychomotor performance once at 14:00 pm, 15:00 pm, 16:00 pm, 17:00 pm, 19:00 pm and 21:00 pm, for six times in total after taking 10 mg or 15 mg zaleplon and placebo alternately at 13:00 pm. Another six volunteers participated in the vestibular function evaluation with the same experimental design and procedure.

### Data analysis

The sleep data were subjected to one-way analysis of variances (ANOVA) using SPSS 13.0 statistical software. The experimental data of psychomotor performance and the vestibular function evaluations were subjected to repeated-measures analysis of variance with two factors (drug and time). *P* < 0.05 was used as the level of significance for the ANOVAs and for post hoc testing of the significant drug × trial interactions.

## Results

### Sleep process and sleep structure

The results are presented in Table 1. Significant differences in the SL, SE and REM% were observed among the three groups (*F*_(2, 21)_ = 24.05, 29.94 and 22.54, respectively, *P* < 0.01). Compared with the placebo group, the SL in the zaleplon 10 mg group was significantly decreased (*P* < 0.05). In the zaleplon 15 mg group, the SL was significantly decreased, and the SE and REM% were remarkably increased (*P* < 0.01) compared with the placebo group and the zaleplon 10 mg group. In the zaleplon 15 mg group, the percentage of slow wave sleep in N3 was significantly decreased (*P* < 0.01) compared with the zaleplon 10 mg group.

**Table 1:** Effects of zaleplon at two doses on sleep process and sleep structure ($\overline{x}\pm s$, *n* = 8)

Index	Placebo group	Zaleplon 10 mg group	Zaleplon 15 mg group
Sleep process			
Sleep latency (min)	27.50 ± 10.92	11.19 ± 2.05^*^	5.75 ± 2.12^**^
Sleep efficiency (%)	68.71 ± 6.04	69.63 ± 3.69	87.40 ± 6.22^**∆∆^
Sleep structure			
N1 (%)	32.58 ± 5.68	29.52 ± 3.77	31.58 ± 5.26
N1 (%)	45.86 ± 8.98	42.37 ± 6.68	37.95 ± 10.16
N3 (%)	15.21 ± 7.01	20.96 ± 7.87	12.71 ± 3.97^∆^
REM (%)	6.35 ± 3.44	7.16 ± 2.76	17.76 ± 4.87^**∆∆^

Maximum time in bed was 270 min. ^*^ *P* < 0.05, ^**^ *P* < 0.01 compared with placebo group; ^△^ *P* < 0.05, ^∆∆^ *P* < 0.01 compared with zaleplon 10 mg group

### Subjective evaluations of sleep quality and sleepiness after awakening

As shown in Table 2, there were significant differences in the scores of subjective SQS and SDS among the three groups (*F*_(2, 21)_ = 13.043 and 13.233, respectively, *P* < 0.01). Compared with the placebo group, the score of the subjective SQS was significantly enhanced in the zaleplon 10 mg and zaleplon 15 mg groups after awakening (*P* < 0.01). Compared with the placebo group, the scores of the subjective SDS were also significantly enhanced in the zaleplon administered groups (*P* < 0.05). Compared with the zaleplon 10 mg group, the subjective SDS score was markedly increased after ingestion of zaleplon 15 mg (*P* < 0.05). However, there were no obvious differences of subjective sleepiness among the three groups after awakening.

**Table 2:** Effects of zaleplon at two doses on subjective sleep quality and sleepiness ($\overline{x}\pm s$, *n* = 8)

Index	Placebo group	Zaleplon 10 mg group	Zaleplon 15 mg group
Scores of SQS	3.75 ± 1.04	5.38 ± 0.92^**^	6.00 ± 0.76^**^
Scores of SDS	3.25 ± 1.04	4.62 ± 1.41^*^	6.12 ± 0.83^**∆^
Scores of SSS	2.38 ± 0.92	2.12 ± 0.64	2.38 ± 0.92

The subjective evaluations were carried out after awakening. ^*^ *P* < 0.05, ^**^ *P* < 0.01 compared with placebo; ^∆^ *P* < 0.05 compared with zaleplon 10 mg group

### Psychomotor performance

There were no significant differences for the correct rate and maintaining the rate of the dual tasks (F_(2, 21)_ = 0.047 and 0.372, *P* > 0.05, Table 3) among the three groups. Compared with the placebo, the reaction times of red, green and yellow lights (*F*_(2, 21)_ = 0.113, 0.813 and 0.675, *P* > 0.05) were slightly prolonged after taking zaleplon 10 mg and 15 mg; however, there were no significant differences among the groups. Compared with the placebo, the CFF was slightly decreased after taking zaleplon 10 mg and 15 mg, although no significant differences were observed among the three groups (*F*_(2, 21)_ = 0.327, *P* > 0.05).

**Table 3:** Effects of zaleplon at two doses on the correct rate of dual task ($\overline{x}\pm s$, *n* = 8)

Group	Baseline	Time after taking drug					
		1 h	2 h	3 h	4 h	6 h	8 h
Placebo	92.99 ± 3.27	90.78 ± 7.15	91.86 ± 5.06	89.83 ± 6.88	90.55 ± 3.59	90.79 ± 4.34	90.86 ± 5.53
Zaleplon 10 mg	88.54 ± 8.58	88.59 ± 6.53	89.66 ± 4.33	90.09 ± 4.37	93.31 ± 4.92	92.34 ± 5.24	92.76 ± 5.55
Zaleplon 15 mg	87.01 ± 6.31	92.38 ± 3.34	88.82 ± 4.38	91.65 ± 6.82	91.37 ± 4.57	90.48 ± 4.21	91.76 ± 6.21

The baseline of the placebo group served as 100 %; *F*_(2, 21)_ = 0.047, *P* > 0.05

### Vestibular function

**Fig. 1:**
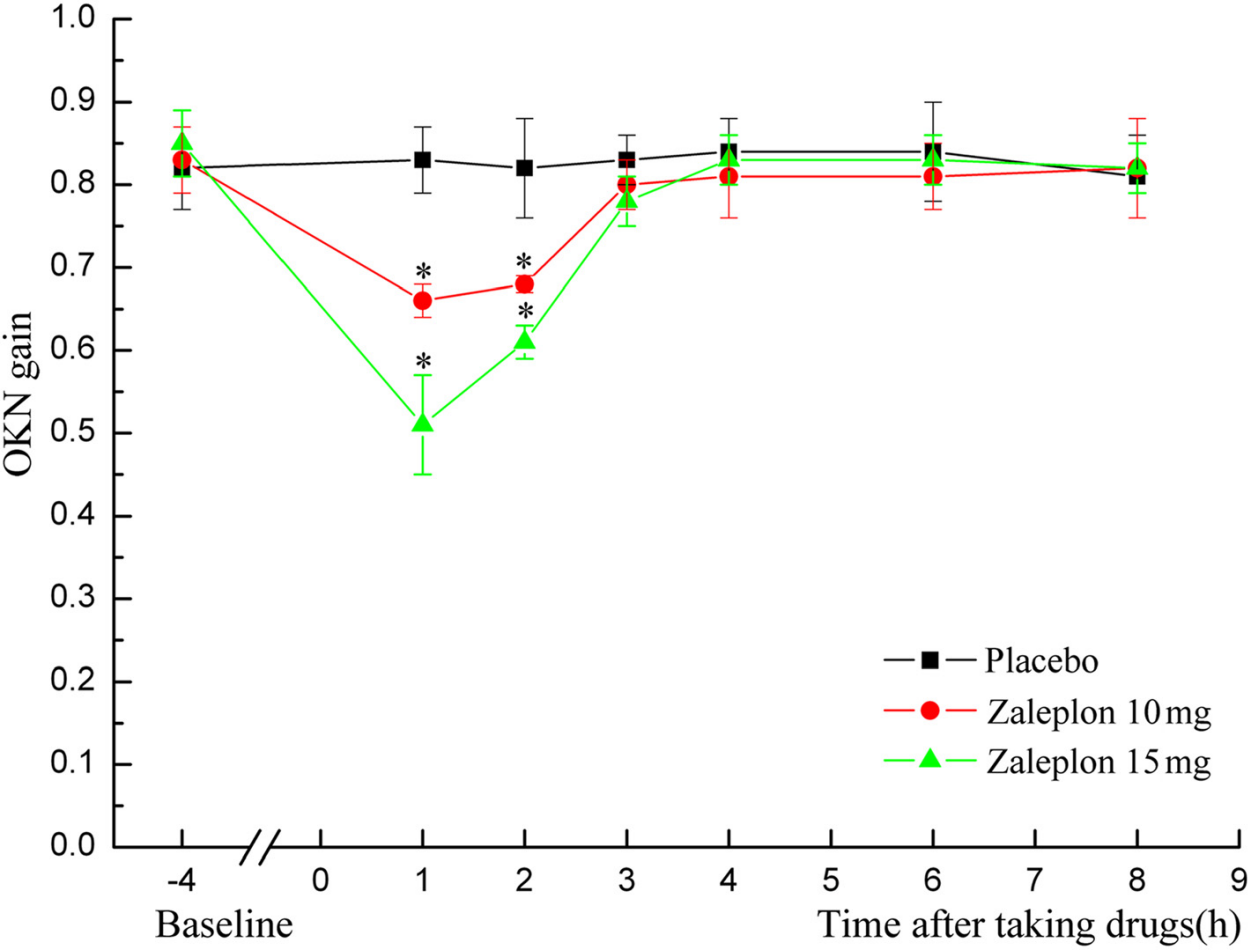
Effects of zaleplon on optokinetic nystagmus (OKN) gain. ^*^ *P* < 0.05 compared with placebo

Compared with the placebo group, the OKN gain was significantly decreased in the two dose groups of zaleplon (*F*_(2, 15)_ = 10.81, *P* < 0.05, Fig. 1) and restored to normal 3 h after drug ingestion; the same trend was observed for the VOR gain (*F*_(2, 15)_ = 39.64, *P* < 0.05), whereas the VVOR and VOR-Fix gains did not change significantly at any times points after ingestion of zaleplon 10 mg and 15 mg. Every time subjects in the three groups gazed at the fixed light, vestibular nystagmus was completely inhibited. The nystagmogram showed a nearly single line, which indicates that fixation suppression was complete.

### Other adverse effects observation

Compared with the placebo group and the zaleplon 10 mg group, zaleplon at a dose of 15 mg caused significant drowsiness up to 2 h postdose during the psychomotor performance and vestibular test sessions. No other adverse effects were observed or reported after taking 10 mg or 15 mg of zaleplon.

## Discussion

Zaleplon is commonly administered at 10 mg oral doses, although some experiments showed that this dosage had no significant sleep-inducing effects. Drake et al. [14] found that zaleplon 10 mg produced a decrease in latency to persistent sleep that was comparable with that of triazolam 0.25 mg but did not produce significant increases in total sleep time over placebo for patients with chronic primary insomnia. Simons et al. [8] observed the usefulness of temazepam (20 mg) and zaleplon (10 mg) to improve 4.5 h of sleep in the afternoon and found that the use of zaleplon had no significant advantage over placebo. The results of this study showed that zaleplon 15 mg provided significantly superior sleep compared with zaleplon 10 mg or the placebo under noise interference.

The methods of evaluating the effects of drugs on central functions mainly include cognitive performance, CRT, CFF, some subjective psychological scales and others [15, 16]. Paul et al. [17] compared the effect of a single dose of zaleplon (10 mg), zopiclone (7.5 mg), temazepam (15 mg), and melatonin (6 mg) on psychomotor performance and quantified the post-ingestion time required for return to normal performance. The results showed that melatonin was superior to zaleplon in causing no effect on performance. The remaining drugs listed in increasing order of performance effect duration were zaleplon (2.25–3.25 h), temazepam (4.25–6.25 h), and zopiclone (3.25–5.25 h). Whitmore et al. [18] reported that zaleplon (10 mg), when used as a daytime sleep aid, causes drowsiness (and related symptoms) up to 3 h postdose and might affect task performance, especially more complex tasks, for at least 2–3 h postdose. Verster et al. [19] observed the effects of a middle-of-the-night administration of zaleplon 10 or 20 mg, zolpidem 10 or 20 mg, or placebo on driving ability 4 h after administration and on memory and psychomotor performance 6 h after administration. The results showed that zaleplon (10 and 20 mg) was a safe hypnotic devoid of next-morning residual impairment when used in the middle of the night. However, a higher number of adverse events were observed with the 40 and 60 mg doses of zaleplon compared with triazolam (0.25) and placebo [14]. Therefore, the present results are in accordance with the results of previous studies [17–19], although some differences exist due to the variances of participants and experimental conditions.

Vestibular function is very important for spatial orientation and anti-air sickness of aircrew and some other special military personnel. In general, the vestibular optokinetic reflex of human beings is easily regulated by the cerebral cortex; OKN is mainly influenced by the cerebral cortex and brain stem; VOR-Fix suppression is influenced by the cerebellum; and the vestibular nuclei, cerebellum and reticular structure in the brain stem play important roles in the regulation of the visual-vestibular optokinetic reflex. A variety of drugs, especially barbiturates, antihistamines, anticonvulsants and alcohol, could induce functional excitation or inhibition of the central nervous system and influence vestibular function [13]. Collins [20] observed that under stimulation with angular acceleration, d-amphetamine significantly increased nystagmus and enhanced the “rotation” experience of subjects during 55 h of sleep deprivation. In our previous study, OKN gain in the modafinil group was increased significantly during 24 h of sleep deprivation, and the drug’s effect was maintained for 1–7 h, which might be related to its central stimulation effects and pharmacokinetic features [21, 22]. In this study, the OKN and VOR gains were lower in the two zaleplon dose groups (*P* < 0.05) and restored to normal 3 h after drug ingestion. These results are in accordance with the experimental observation of its effects on psychomotor performance, which could be explained by its minor central inhibitive effects and very short half-life [23]. In addition, some related literature reported that zaleplon did not influence performance under altitude environment and did not influence driving ability [24–26]. Based on the hypnotic efficiency and the adverse effects on central function, zaleplon is an ideal hypnotic for aircrew and some other military personnel.

## Conclusion

Modern military operations might require pharmacological methods to sustain alertness and facilitate sleep to maintain operational readiness. In operations with very limited sleep windows, hypnotics with a very short half-life might be used. Based on the hypnotic efficiency and the adverse effects on central function, zaleplon is an ideal hypnotic for aircrew and some other military personnel, and the optimal single dosage for sleep induction could be increased from the routine 10 mg to 15 mg.

## Abbreviations

CFF: critical flicker fusion
CRT: choice reaction time
GABA: γ-aminobutyric acid
OKN: optokinetic nystagmus
PSG: polysomnography
REM: rapid eye movement
SDS: sleep depth scale
SE: sleep efficiency
SL: sleep latency
SQS: sleep quality scale
SSS: the stanford sleepiness scale
VOR: vestibular ocular reflex
VOR-Fix: vestibular ocular reflex fixation suppression
VVOR: visual-vestibular ocular reflex
